# Negative Feedback Regulation of Wnt4 Signaling by EAF1 and EAF2/U19

**DOI:** 10.1371/journal.pone.0009118

**Published:** 2010-02-09

**Authors:** Xiaoyang Wan, Wei Ji, Xue Mei, Jiangang Zhou, Jing-xia Liu, Chengchi Fang, Wuhan Xiao

**Affiliations:** Key Laboratory of Biodiversity and Conservation of Aquatic Organisms, Institute of Hydrobiology, Chinese Academy of Sciences, Wuhan, People's Republic of China; Texas A&M University, United States of America

## Abstract

Previous studies indicated that EAF (ELL-associated factor) family members, EAF1 and EAF2/U19, play a role in cancer and embryogenesis. For example, EAF2/U19 may serve as a tumor suppressor in prostate cancer. At the same time, EAF2/U19 is a downstream factor in the non-canonical Wnt 4 signaling pathway required for eye development in *Xenopus laevis*, and along with EAF1, contributes to convergence and extension movements in zebrafish embryos through Wnt maintenance. Here, we used zebrafish embryos and mammalian cells to show that both EAF1 and EAF2/U19 were up-regulated by Wnt4 (Wnt4a). Furthermore, we found that EAF1 and EAF2/U19 suppressed Wnt4 expression by directly binding to the *Wnt4* promoter as seen in chromatin immunoprecipitation assays. These findings indicate that an auto-regulatory negative feedback loop occurs between Wnt4 and the EAF family, which is conserved between zebrafish and mammalian. The rescue experiments in zebrafish embryos showed that early embryonic development required the maintenance of the appropriate levels of Wnt4a through the feedback loop. Others have demonstrated that the tumor suppressors p63, p73 and WT1 positively regulate Wnt4 expression while p21 has the opposite effect, suggesting that maintenance of appropriate Wnt4 expression may also be critical for adult tissue homeostasis and prevention against tumor initiation. Thus, the auto-regulatory negative feedback loop that controls expression of Wnt4 and EAF proteins may play an important role in both embryonic development and tumor suppression. Our findings provide the first convincing line of evidence that EAF and Wnt4 form an auto-regulatory negative feedback loop *in vivo*.

## Introduction

Evidence strongly supports the involvement of EAF (ELL-associated factor) family members in cancer, particularly in prostate cancer and acute myeloid leukemia [1], [2]. Studies initially identified EAF1 and EAF2/U19 (Up-regulated gene 19) through their association with ELL (Eleven-nineteen lysine-rich leukemia), a fusion partner of MLL in the t (11; 19) (q23; p13.1) chromosomal translocation associated with acute myeloid leukemia [3], [4], while an independent study simultaneously identified EAF2/U19 through its up-regulation by androgen in the rat prostate [5]. ELL binds to RNA polymerase II and acts as a transcription elongation factor whose targeted deletion leads to embryonic lethality in mice [6], [7]. By binding to ELL, both EAF1 and EAF2/U19 efficiently stimulate ELL elongation activity [8]. Luo et al. found that the ability of ELL-MLL to induce leukemogenesis requires the EAF1-interaction domain in ELL [2]. As well, we previously showed through *in vitro* and *in vivo* functional assays that EAF2/U19 serves a tumor suppressive role in prostate cancer [1], [9], [10]. EAF2/19 inhibits xenograft prostate tumor growth and is down-regulated in prostate cancer cell lines. In addition, human advanced prostate cancer specimens exhibit EAF2/U19 down-regulation, allelic loss, promoter hypermethylation, and homozygous deletion [1]. Consistent with its potential tumor suppressive role in the human, *Eaf2/U19*-knockout mice develop lung adenocarcinoma, hepatocellular carcinoma, B-cell lymphoma, and high-grade prostate intraepithelial neoplasia [10]. Moreover, we recently showed that EAF2/U19 binds to and stabilizes the classic tumor suppressor, pVHL [11]. While these findings link EAF proteins to cancer, the molecular mechanisms underlying this involvement remain unclear.

In addition to cancer, for some species, EAF proteins may also play an important role in embryogenesis through non-canonical Wnt signaling [12]. *Eaf2/U19* is not required for embryogenesis, as the intercross between heterozygous mice yielded phenotypically normal offspring with the expected 1∶2∶1 genotypic ratios [10]. However, in *Xenopus laevis,* normal eye development requires the expression of the *eaf2/u19* gene, a target of non-canonical Wnt4 signaling [12]. Wnt4 is a member of the Wnt family of secreted glycoproteins important in tissue and organ organization during development [13]. Some members of the Wnt family function by stabilizing β-catenin; this pathway is referred to as canonical signaling. Other Wnt proteins, such as Wnt4, function independently of β-catenin and thus are involved in non-canonical signaling. Evidence supports the involvement of Wnt4 in embryogenesis. *Xenopus*, mouse and chicken embryos express Wnt4 in distinct expression domains in neural tissues as well as the developing excretory system [12], [14]. Similarly, loss-of-function studies have shown kidney organogenesis in mouse and *Xenopus* requires Wnt4 [12], [15].

Zebrafish have two Wnt4 isoforms: Wnt4a (Wnt4) and Wnt4b. Ungar and colleagues cloned *wnt4a*, initially named *wnt4*, from zebrafish using an orthologous gene search. Based on the phenotypes of zebrafish and *Xenopus* embryos injected with synthetic mRNA, zebrafish *wnt4a* and *Xenopus* Xwnt-5A appear to share a similar function, distinct from that of wnt1, Xwnt3A and Xwnt8 [16]. Little is known regarding the function of *wnt4b*, a duplicate gene of *wnt4a*. However, work from Liu et al. has demonstrated that the zebrafish floor plate exclusively expresses *wnt4b* mRNA and that sonic hedgehog and gli-2 zebrafish mutants alter this expression [17]. In non-canonical Wnt signaling, zebrafish Wnt4a appears to be much closer to that of mammalian Wnt4 [18].

During function assays for the *Eaf* gene family in zebrafish embryogenesis, we found that Eaf1 and Eaf2/U19 could regulate gene expression of the non-canonical Wnt signaling ligands, *wnt5*, *wnt11*, *wnt11r* and *wnt4* [19]. Given that EAF2/U19 is downstream of Wnt4 in *Xenopus laevis* [12], we hypothesized that a regulation loop may exist between the EAF family and the non-canonical Wnt signaling ligand, Wnt4. Using zebrafish embryos and mammalian cell line 293, we found that Wnt4 up-regulated both EAF1 and EAF2/U19, while both EAF1 and EAF2/U19 efficiently suppressed Wnt4 expression, supporting the existence of an auto-regulatory negative feedback loop.

## Materials and Methods

### Maintenance of Fish Stocks and Embryo Collection

Breeding wild-type zebrafish (*Danio rerio*) (AB) were maintained and embryos rose under standard library conditions approved by the research committee of Institute of Hydrobiology, CAS.

### Cell Lines and Plasmid Construction

HEK 293 cells were obtained from ATCC. Cells were maintained in Dulbecco Modified Eagle Medium (Gibco) with 10% fetal bovine serum (FBS, Hyclone), 1% Glutamine, and 1% penicillin-streptomycin (Hyclone) at 37°C in a humidified atmosphere containing 5% CO_2_.

Human *Wnt4* cDNA, provided by Andrew McMahon, was cloned into the vector pCGN-HAM (provided by William Tansey). Human *EAF1* and *EAF2/U19* were also subcloned into pCGN-HAM. Zebrafish *eaf1*, *eaf2/u19* and *wnt4a* were RT-PCR amplified and subcloned into both the Psp64 poly(A) vector (Promega) and the CMV-Myc vector (Clontech) (The PCR primer sequences will be provided upon request). Human *Wnt4* promoter luciferase constructs (pGL2-Basic) (2.6kb and 1.2 kb) were provided by Paul Goodyer. The promoter regions for zebrafish *eaf1* (−1288−+69), zebrafish *eaf2/u19* (−663−+337), human *EAF2/U19* (−3222−+436) and human *EAF1* (+616−+262) were PCR amplified and subcloned into a pGL3-Basic vector (Promega). All constructs were verified by sequencing. The cell line transient transfections were carried out using Lipofectamine 2000 (Invitrogen).

### Luciferase Reporter Assays

For zebrafish *eaf1* and *eaf2/u19* promoter assays, zebrafish embryos were injected with the indicated amounts of vectors and the *Renilla* luciferase reporter as an internal control. For human *Eaf1* and *Eaf2/U19* promoter assays, 293 cells grown on 24-well plates were transfected with the indicated amounts of vectors and the *Renilla* luciferase reporter as an internal control, using Lipofectamine 2000. The luciferase activity in embryos lysates or cell extracts was determined 24 hours post fertilization (hpf) or 24–30 hours post transfection using the Dual-luciferase Reporter Assay System (Promega). The relative light units were measured using a luminometer (Sirius, Zylux Corporation, Oak Ridge, TN). Data were normalized by *Renilla* luciferase. Data are reported as mean ± SEM of three independent experiments performed in triplicate. The statistical analysis (paired t-test) was performed using GraphPad Prism 5.

### Morpholino Injection, mRNA Synthesis and *In Situ* Hybridization

Morpholinos against zebrafish *eaf1* (EAF1-MO1) and *eaf2/u19* (EAF2-MO1) were described previously [19]. Morpholino against zebrafish wnt4a (Wnt4-MO) was also described previously [18]. MRNA synthesis and whole mount *in situ* hybridization were performed as described previously [19].

### Semi-Quantitative RT-PCR

Using the RNeasy Mini Kit (Qiagen), we isolated total RNA from wild-type zebrafish embryos and embryos that had been injected with *wnt4a* mRNA, GFP mRNA (100 pg), zebrafish *eaf1* mRNA (100 pg), zebrafish *eaf2/u19* mRNA(100 pg), EAF1-MO1(8 ng), Eaf2-MO1(8 ng) or Standard Morpholino (STD-MO) (8 ng). The RevertAid™ First Strand cDNA Synthesis Kit (Fermentas, Burlington, Ontario) and random primers were used to reverse transcribe 2 µg RNA. As a template for the semi-quantitative RT-PCR, we used first strand cDNA corresponding to 50 ng of total RNA. Semi-quantitative PCR was performed in the presence of SYBR green using a Chromo4™ Detector for the PTC DNA Engine™ System (Bio-Rad). All PCR reactions were run in triplicate and repeated at least three times. Differences were calculated according to the ΔΔ Ct relative quantization method using the β-actin gene to calibrate. The primers for zebrafish *wnt4a* were 5′- TTCAGGCTCCTGGGAAGTCAAGA -3′ and 5′- TGCGGCTTGAATTGTGAGTTTCG -3′. The primers for zebrafish *eaf1* were 5′- ATGATCGAGCAGATGAGCAGCAGT-3′ and 5′- TGTGTTCATCAGCTGGTTGTTGCC-3′. The primers for zebrafish *eaf2* were 5′- TGGAAGGATCCACAGCACCAGTTA-3′ and 5′- TGCTCCAGCCGTGACTGAATCTTA-3′. The primers for zebrafish β-actin gene included 5′- CACTGTGCCCATCTACGAG -3′ and 5′- CCATCTCCTGCTCGAAGTC -3′. We used GraphPad Prism 5 for statistical analysis (paired t-test).

### Antibodies and Western Blot Analysis

Polyclonal antibodies against human EAF1 and EAF2/U19 were raised in rabbit. Briefly, full length human EAF1 (aa 1–268) and EAF2/U19 (aa 1–260) were cloned into the pET-32a vector (Novagen) and then the vectors transformed in the *Escherichia coli* strain BL21 (DE3). The His-tagged EAF1 and EAF2/U19 proteins were induced by 1 mM isopropyl b-D-thiogalactoside, run through a column filled with His.Bind Resin (Novagen) and then eluted in buffer consisting of 1 M imidazole, 500 mM NaCl, and 20 mM Tris-HCl (pH 7.9). The purified fusion protein was dialyzed against Tris-buffered saline (TBS; pH 7.5) overnight. Rabbits were immunized three times with the purified His-tagged EAF1 or EAF2/U19 mixed with Freund adjuvant. Anti-α-tubulin monoclonal antibody was purchased from Upstate. Anti-β-actin antibody, anti-GAPDH and anti-myc antibody were purchased from Santa Cruz. Anti-HA (monoclonal) was purchased from Covance Research Products (Cumberland, Virginia).

Western blots were performed as described previously [9]. A FujiFilm LAS4000mini luminescent image analyzer was used to photograph blots. Quantitative analysis was performed using a Multi Gauge v3.0 in addition to a LAS4000mini analyzer. We used GraphPad Prism 5 for graph preparation and statistical analysis (paired t-test).

### Chromatin Immunoprecipitation

Chromatin immunoprecipitation (ChIP) assays were performed according to a modified protocol from the acetyl-histone H3 immunoprecipitation kit manual (Upstate). 293 cells were cross-linked with 1% formaldehyde in PBS for 2 min at room temperature, washed three times with cold PBS, and then scraped off the plate. Scraped cells were lysed with lysis buffer that included proteinase inhibitors and 1 mM dithiothretol. Cell lysates were sonicated to break down chromatin into pieces 200–500 base pairs in length. Chromatin was diluted 1∶10, and pre-cleared with protein A agarose beads (Upstate) for 1 h at 4°C. Approximately 3% of the chromatin from each sample was saved as input and the rest used for immunoprecipitation with polyclonal rabbit anti-EAF1, anti-EAF2/U19 or preimmune serum as a control at 4°C overnight. Immunoprecipitated antibody–protein–DNA complexes were collected by protein A agarose beads at 4°C for 1 h and then extensively washed. The complex was eluted and reverse cross-linked at 65°C for 6 h, and then proteinase K added for a further incubation at 45°C for 1 h. DNA samples were extracted using phenol:chloroform. PCR was performed for 35 cycles with the following cycling condition: 94°C, 30 s; 53°C, 30 s; 72°C, 1 min. The reactions included primers for the human *Wnt4* promoter (5′- CCTGGTAGCCTGGCAAATCTTCC -3′ and 5′- TCCCATGGTCTTCCCTCCTTGTGA -3′) or for the promoter region of β-actin (5′-CAGGGCGTGATGGTGGGCA-3′ and 5′- CAAACATGATCTGGGTCATCTTCTC -3′), which was used as internal control. The locations of primers are indicated.

### Northern Blot Analysis

293 cells were transfected with the pCGN-HAM empty vector, or vectors expressing HA-EAF1 or HA-EAF2/U19. Total RNA was isolated with Trizol reagent (Invitrogen). Electrophoresis, transfer and hybridization were performed as described previously [5]. The membrane was probed using synthesized oligos corresponding to human Wnt4 (5′-atggccttggacgtcttgttgcatgt-3′) and human β-actin (5′-atgtgcaatcaaagtcctcggccaca-3′) labeled with biotin at the 3′ end. The signal was detected using the North2South Nucleus Labeling and Detection Kit (Pierce). Photography and data analysis were done as described for the Western blot analysis.

## Results

### Zebrafish Eaf1 and Eaf2/u19 Are Wnt4a Downstream Factors

We had shown that during zebrafish embryogenesis, *eaf1* and *eaf2/u19* mediated convergence and extension movements as well as midline convergence of organ primordia by regulating expression levels of the non-canonical Wnt ligands, *wnt5*, *wnt11* and *wnt11r* [19]. This corroborated previous studies that had established roles for *wnt5* and *wnt11* in regulating convergence and extension movements [20], [21], and *wnt11* and *wnt11r* in midline convergence of organ primordia [18]. To gain a more complete picture of Eaf1 and Eaf2/U19-mediated regulation of non-canonical Wnt ligands, we had also evaluated the expression of *wnt4a* mRNA in the Eaf1 and Eaf2 morphants. Surprisingly, in contrast to other non-canonical ligands (*wnt5*, *wnt11* and *wnt11r*), morpholino-mediated Eaf1 and Eaf2/U19 knockdown resulted in an increase in *wnt4a* expression. Given that others had also shown that *eaf2/u19* is downstream of Wnt4 in *Xenopus* [12], we set forth to further explore the relationship between EAF proteins and Wnt4a.

**Figure 1:**
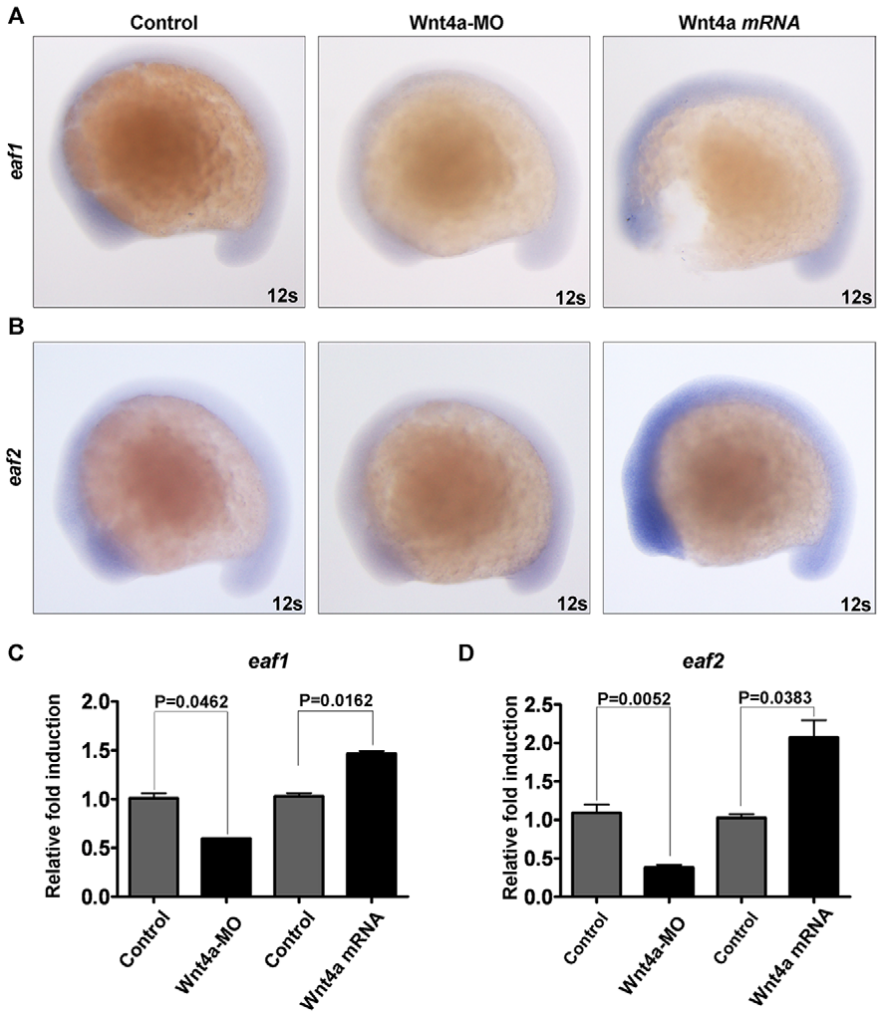
Both zebrafish *eaf1* and *eaf2/u19* genes are downstream factors of zebrafish Wnt4a. (**A**) Zebrafish embryos were injected with Wnt4-MO and *wnt4a* mRNA and the levels of zebrafish *eaf1* mRNA determined by whole-mount *in situ* hybridization at 12-somite stage. (**B**) Zebrafish embryos were injected with Wnt4-MO and *wnt4a* mRNA and the levels of zebrafish *eaf/u19* mRNA determined by whole-mount *in situ* hybridization. 12s, 12 somites. (**C**) Semi-quantitative RT-PCR analysis of *eaf1* expression in zebrafish embryos (12 somites) injected with Wnt4a-MO and *wnt4a* mRNA. (**D**) Semi-quantitative RT-PCR analysis of *eaf2/u19* expression in zebrafish embryos (12 somites) injected with Wnt4a-MO and *wnt4a* mRNA.

**Figure 2:**
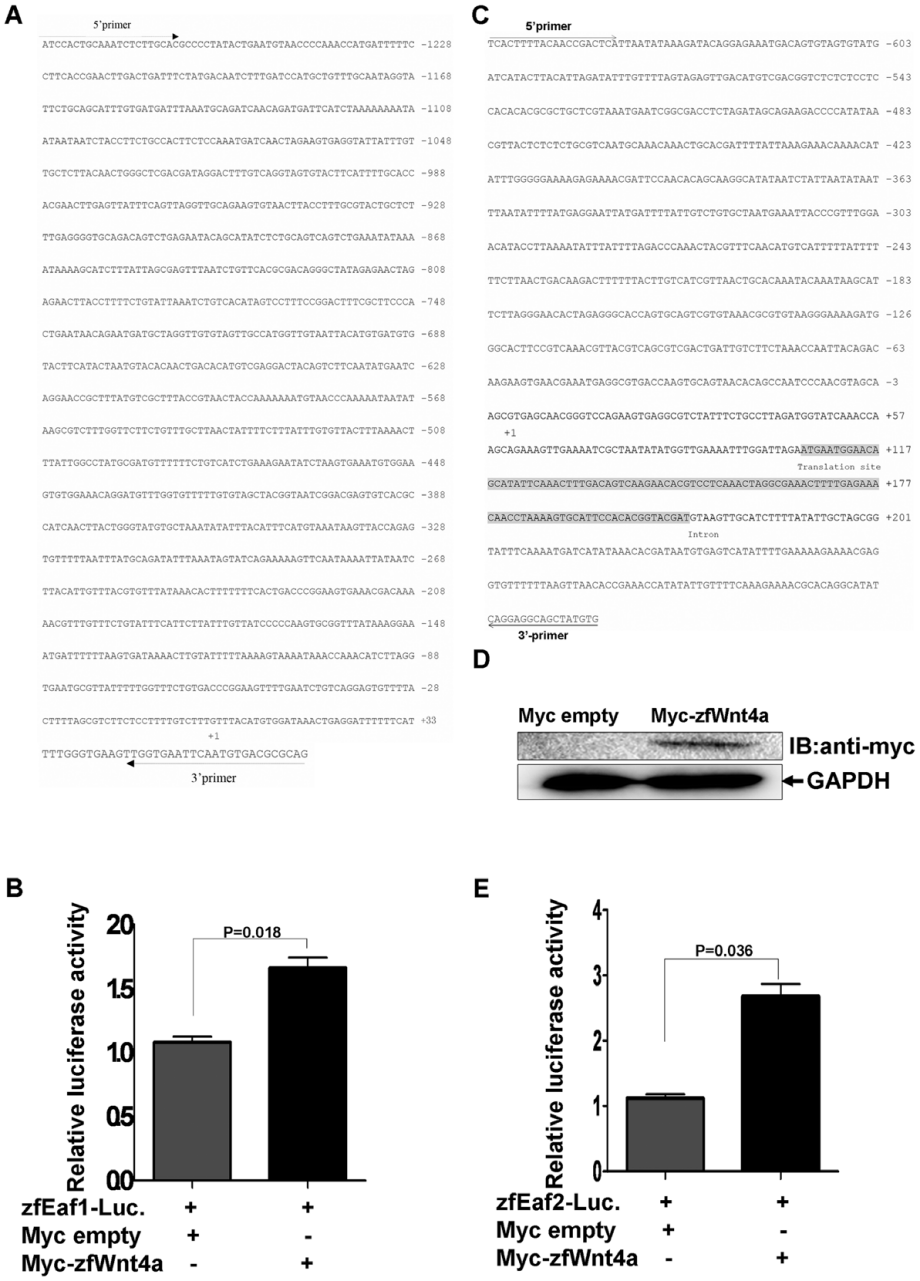
Zebrafish *wnt4a* can activate the zebrafish *eaf1* and *eaf2/U19* promoters. (**A**) The sequence of the 1.2 kb zebrafish *eaf1* promoter and the positions of primers used for subcloning. The transcriptional initial site is indicated by +1. (**B**) Zebrafish embryos were co-injected with the *eaf1* promoter reporter and either a control vector or a vector expressing Myc-Want4a. The luciferase activity was normalized to *Renilla* and reported as the mean ± standard deviation (SD). (**C**) The sequence of the 1.0 kb zebrafish *eaf2/u19* promoter and the primers used for subcloning. The transcriptional initial site is indicated by +1. (**D**) The expression of Myc-Wnt4a was verified by Western blot using an anti-Myc antibody. (**E**) Zebrafish embryos were co-injected with the *eaf2/u19* promoter reporter and either a control vector or a vector expressing Myc-Wnt4a. The luciferase activity was normalized to *Renilla* and reported as the mean ± SD.

To investigate the relationship between Wnt4a and the Eaf family we first injected zebrafish embryos with *wnt4a* mRNA and then performed whole-mount *in situ* hybridization to determine the *eaf1* and *eaf2/u19* expression patterns. Consistent with the observation of *eaf2/u19* up-regulation by wnt4 in *Xenopus*, ectopic *wnt4a* mRNA increased *eaf2/u19* mRNA levels in the zebrafish embryo at 6-somite (Data not shown), 12-somite (Fig. 1B, the third column from left to right) and 18-somite stages (Data not shown). Ectopic *wnt4a* mRNA also led to an increase in *eaf1* expression at 12-somite stage (Fig. 1A, the third column from left to right). On the contrary, the expression of *eaf1* and *eaf2/u19* was down-regulated in the embryos injected with Wnt4a antisense morpholino—Wnt 4a-MO [18] (Fig. 1A and 2B, the second columns from left to right). Semi-quantitative RT-RCR confirmed the ability of Wnt4a to up-regulate *eaf1* and *eaf2/u19* expression significantly as well as showed that Wnt4a induced more *eaf2/u19* expression than *eaf1* (Fig. 1C and 1D, two columns at the right). Consistently, the knockdown of Wnt4a by Wnt4a-MO injection caused down-regulation of both *eaf1* and *eaf2/u19* (Fig. 1C and 1D, two columns at the left).

To further verify the up-regulation of Eaf family members by Wnt4a, we did promoter assays. We cloned approximately 1.2 kb of the zebrafish *eaf1* promoter and 1.0 kb of the *eaf2/u19* promoter into a pGL3-Basic vector (Fig. 2A & 2C). The resultant *eaf1* and *eaf2/u19* promoter reporter constructs were then co-injected with either a Myc-empty vector or Myc-tagged *wnt4a* vector into zebrafish embryos, along with a *Renilla* control. After 24 hpf, we did luciferase assays using embryo lysates. Introduction of the *wnt4a* vector increased the transcriptional activity of the *eaf1* promoter by about 1.8-fold (Fig. 2B) and the *eaf2/u19* promoter by about 3-fold (Fig. 2E), consistent with the results obtained by whole-mount *in situ* hybridization and RT-PCR analysis (Fig. 1A & 1B). Western blot analysis confirmed Wnt4a protein expression (Fig. 2D). To rule out the possibility that Wnt4a non-specifically induced luciferase expression, we co-injected the pGL3-Basic vector with either the Myc-empty or Myc-tagged *wnt4a* vector. Wnt4a did not affect the baseline level of luciferase activity, further supporting the ability of Wnt4a to specifically induce *eaf1* and *eaf2/u19* promoter activity (Data not shown).

### Zebrafish Eaf1 and Eaf2/u19 Knockdown Rescued Embryo Defects Caused by Ectopic Wnt4a

**Figure 3:**
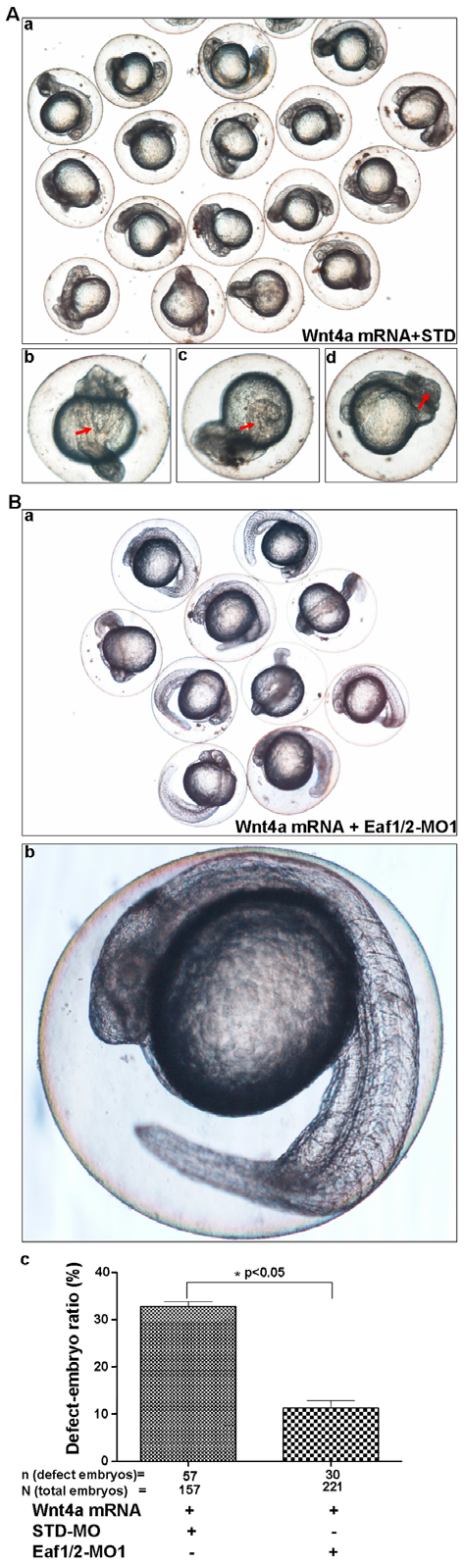
The knockdown of zebrafish Eaf1 and Eaf2/U19 rescues the embryonic defects caused by *wnt4a* over-expression. (**A**) Zebrafish embryos were co-injected with *wnt4a* mRNA (10 pg) and standard morpholino (STD) (8 ng). Defects, indicated by red arrows, included (b) folking notochord, (c) cyclopia, and (d) shortened axis. (**B**) (a) The embryos were co-injected with *wnt4a* mRNA and Eaf1/2-MO1to rescue the phenotype resembles wild-type embryos. (b) An example of a rescued embryo. (c) The efficiency of the Eaf1/Eaf2-MO1-mediated rescue is statistically significant (p<0.05) by counting defective embryos.

To investigate whether the biological effects of Wnt4a are mediated by Eaf1 and Eaf2/U19 *in vivo*, we performed a phenotype analysis in zebrafish embryos. To knockdown expression, we injected embryos with morpholinos against *eaf1* (EAF1-MO1) and *eaf2/u19* (EAF2-MO1) that we had previously shown to be both efficient and specific [19]. As a control, we used the standard morpholino, STD-MO as we had previously demonstrated that injection with this morpholino does not alter zebrafish phenotype [19]. Injection of zebrafish embryos at the one-cell stage with synthetic *wnt4a* mRNA (100 pg) coupled with STD-MO resulted in about 33.9% of the embryos (Fig. 3Aa and Fig. 3Bc) exhibiting cyclopia (Fig. 3Ab), misfolding in the brain (Fig. 3Ac), and an anterior forking notochord (Fig. 3Ac) accompanied with a shortened axis (Fig. 3Ad): these defects resemble those caused by *wnt4a* mRNA injection alone [16]. However, when we included a combination of EAF1-MO1 (4 ng) and EAF2-MO1 (4 ng), only about 10% of the embryos exhibited defects (Fig. 3Ba and 3Bc), while the majority of the embryos appeared morphologically normal (Fig. 3Bb), similar to the wild-type embryos. The ability of EAF1-MO1 and EAF2-MO1 to rescue the phenotype induced by *wnt4a* mRNA was statistically significant (p<0.05) (Fig. 3Bc). These results further support the likelihood that Eaf1 and Eaf2/U19 serve as downstream mediators of Wnt4a signaling, consistent with results obtained in *Xenopus laevis* [12].

### Zebrafish Eaf1 and Eaf2/u19 Suppress Wnt4a Expression

**Figure 4:**
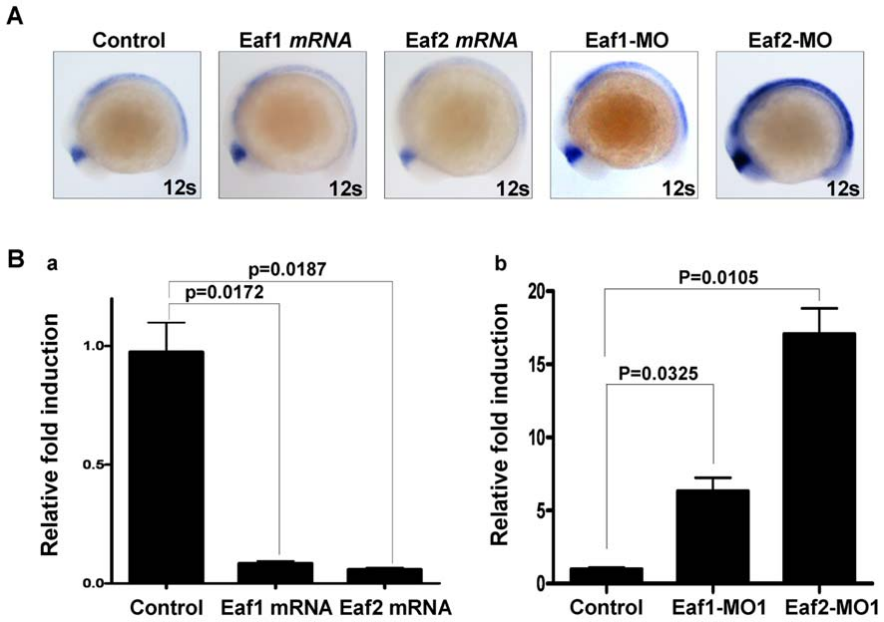
Both zebrafish *eaf1* and *eaf2/u19* suppress zebrafish *wnt4a* expression. (**A**) Whole-mount *in situ* hybridization analysis of zebrafish *wnt4a* expression in embryos injected with *eaf1* mRNA, *eaf2/u19* mRNA, Eaf1-MO and Eaf2-MO at 12-somite stage. The embryos without injection were used as control. (**B**) The expression of zebrafish *wnt4a* mRNA was suppressed by ectopic expression of zebrafish *eaf1* and *eaf2/u19* (a) and up-regulated by knockdown Eaf1 and Eaf2/U19 (b) as revealed by semi-quantitative RT-PCR analysis at 12-somte stage.

Given our previous observation that Eaf1 and Eaf2/U19 knockdown resulted in the up-regulation of *wnt4a* expression [19], we wanted to further evaluate the effect of Eaf1 and Eaf2/U19 on *wnt4a* gene expression. We first determined the *wnt4a* expression pattern during zebrafish embryogenesis by whole mount *in situ* hybridization. The *wnt4a* transcript was initially detected in the dorsocaudal region of the forebrain at the earlier stage of somitogenesis, and then appeared in the dorsal and lateral regions of the caudal hindbrain and the neural plate (Data not shown), the same pattern as reported previously [16]. Next, we injected zebrafish *eaf1* mRNA into embryos at the one-cell stage. At 12-somite stage, the expression of *wnt4a* decreased (Fig. 4A, the second column from left to right) (n = 24/33 at 12-somite stage). Similarly, *wnt4a* mRNA expression decreased in *eaf2/u19* mRNA-injected embryos (Fig. 4A, the third column from left to right) (n = 22/36 at 12-somite stage). Then, we injected antisense morpholinos against either zebrafish *eaf1* or *eaf2/u19* into embryos at the one-cell stage. The expression of *wnt4a* increased by either the *eaf1*-knockdown (Fig. 4A, the forth column from left to right) (n = 29/36 at 12-somite stage) or the *eaf2/u19*-knockdown (Fig. 4A, the fifth column from left to right) (n = 29/29 at 12-somite stage). Interestingly, the *eaf2/u19*-knockdown caused the expression of *wnt4a* increased more significant than that of *eaf1-*knockdown at the 12-somite stage (Fig. 4A, the forth and fifth columns from left to right). To confirm these results, we employed semi-quantitative real-time PCR to analyze levels of *wnt4a* in embryos. The results indicate that injection of either *eaf1* mRNA (p = 0.0172) or *eaf2/u19* mRNA (p = 0.0187) dramatically suppressed *wnt4a* gene expression at 12-somite stage (Fig. 4Ba). As expected, blocking Eaf1 or Eaf2/U19 expression by morpholinos dramatically up-regulated *wnt4a* mRNA levels (Fig. 4Bb). Together, this data suggests that Eaf1 and Eaf2/U19 inhibit *wnt4a* transcription efficiently.

### Mammalian EAF1 and EAF2/U19 Are Wnt4 Downstream Factors

**Figure 5:**
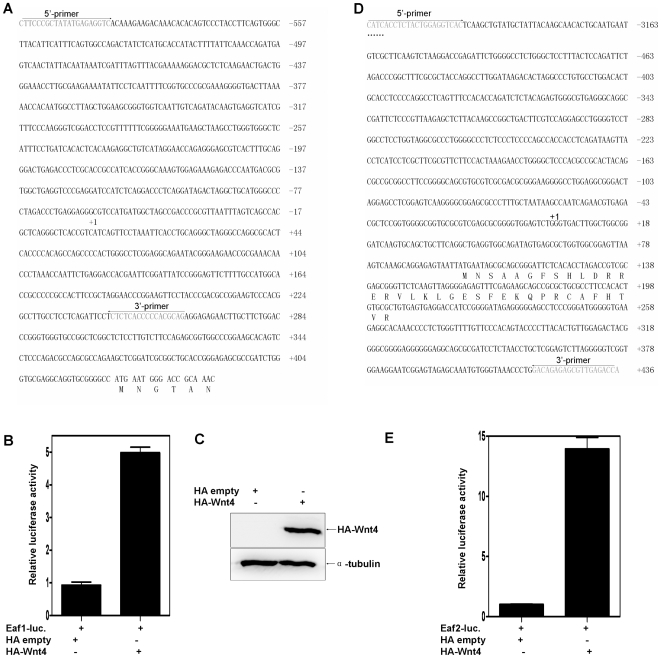
Both human EAF2/U19 and EAF1 are Wnt4 downstream factors. (**A**) The sequence of the 1.0 kb human *EAF1* promoter and the positions of primers used for subcloning. The transcriptional initial site is indicated by +1. (**B**) 293 cells were co-transfected with the *EAF1* promoter reporter and either a control vector or a vector expressing HA-Wnt4. The luciferase activity was normalized to Renilla and reported as the mean ± standard deviation (SD). (**C**) The expression of Wnt4 was verified by Western blot using an anti-HA antibody. (**D**) The partial sequence of the 3.6 kb human *EAF2/U19* promoter and the primers used for subcloning. The transcriptional initial site is indicated by +1. (**E**) .293 cells were co-transfected with the *EAF2/U19* promoter reporter and either a control vector or a vector expressing HA-Wnt4. The luciferase activity was normalized to Renilla and reported as the mean ± SD.

After demonstrating a relationship between the Eaf family and Wnt4a in zebrafish, we next wanted to determine if this relationship exists in mammalian cells; thus, we performed similar assays in the mammalian 293 cell line. As a first step, we cloned the human *EAF1* and *EAF2/U19* promoter regions into the pGL3-basic vector (Fig. 5A and 5D). *EAF1* and *EAF2/U19* reporter constructs were then co-transfected with either an HA-empty vector or HA-tagged *Wnt4* vector along with the *Renilla* control into 293 cells. Wnt4 increased the transcriptional activity of the *EAF1* promoter by about 5-fold (Fig. 5B) and the *EAF2/U19* promoter by about 14-fold (Fig. 5E); similar to what we had seen in the zebrafish *eaf1* and *eaf2/u19* promoter assays (Fig. 2B & E) and consistent with the increase of EAF2/U19 induced by Wnt4 in *Xenopus laevis* [12]. Western blot analysis confirmed Wnt4 protein expression (Fig. 5C). To rule out the possibilitythat Wnt4 non-specifically induced the pGL3-basic vector, we performed a control experiment in which we co-transfected cells with the pGL3-basic vector and either the HA-empty vector or the HA-tagged *wnt4* vector. We found that HA-Wnt4 did not change the level of luciferase activity (Data not shown). These observations indicate that Wnt4 can directly induce the *EAF1* and *EAF2/U19* genes in mammalian cells.

**Figure 6:**
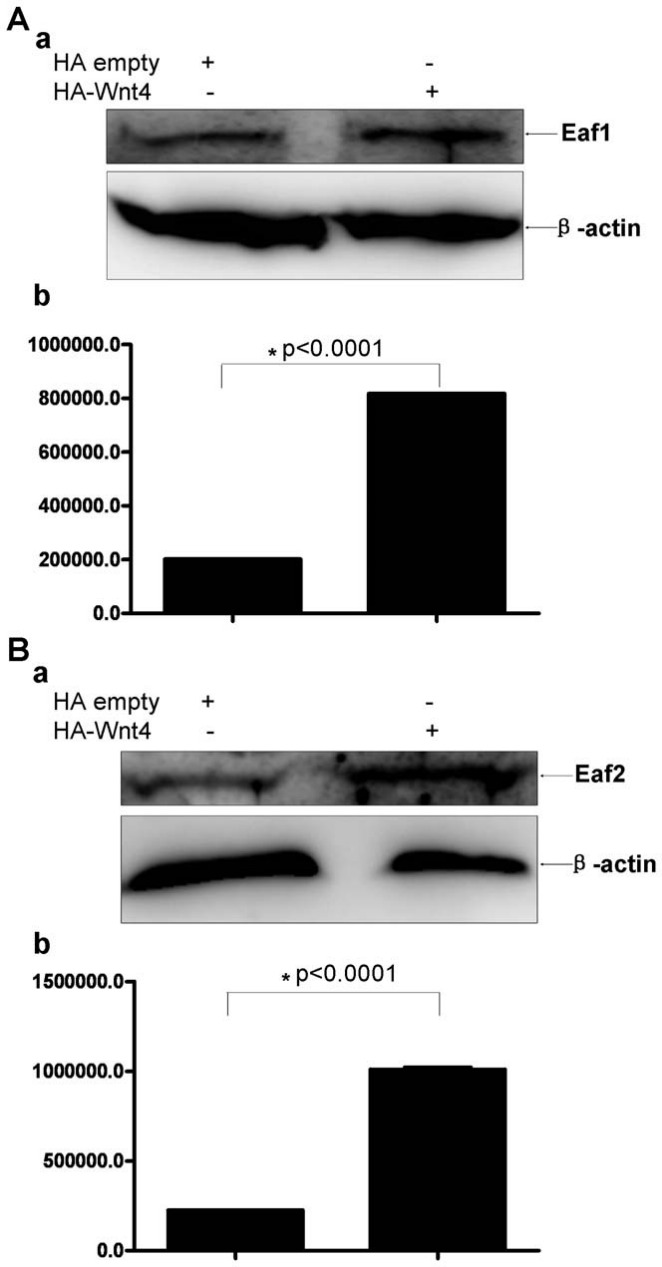
Western blot analysis of human endogenous EAF1 and EAF2/U19 protein expression after the introduction of ectopic *Wnt4* expression in 293 cells. (A) Representative Western blot of EAF1 in 293 cells transfected with a control vector or a vector expressing Wnt4 using a polyclonal antibody against human EAF1. (b) EAF1 expression was normalized to β-actin. (B) Representative Western blot of EAF2/U19 in 293 cells transfected with a control vector or a vector expressing Wnt4 using a polyclonal antibody against human EAF2/U19. (b) EAF2/U19 expression normalized to β-actin.

To determine if Wnt4 over-expression also increased the protein levels of EAF1 and EAF2/U19 in mammalian cells, we conducted Western blot analysis of 293 cells transfected with an HA-empty vector or an expression vector for HA-tagged Wnt4. Anti-EAF1 or anti-EAF2/U19 polyclonal antibodies revealed that Wnt4 induced a dramatic increase in both EAF1 and EAF2/U19 levels (p<0.0001) (Fig. 6A and 6B). Consistent with the promoter assays, Wnt4 induced expression of more EAF2/U19 than EAF1 protein.

### Mammalian EAF1 and EAF2/U19 Suppress Wnt4 Expression

**Figure 7:**
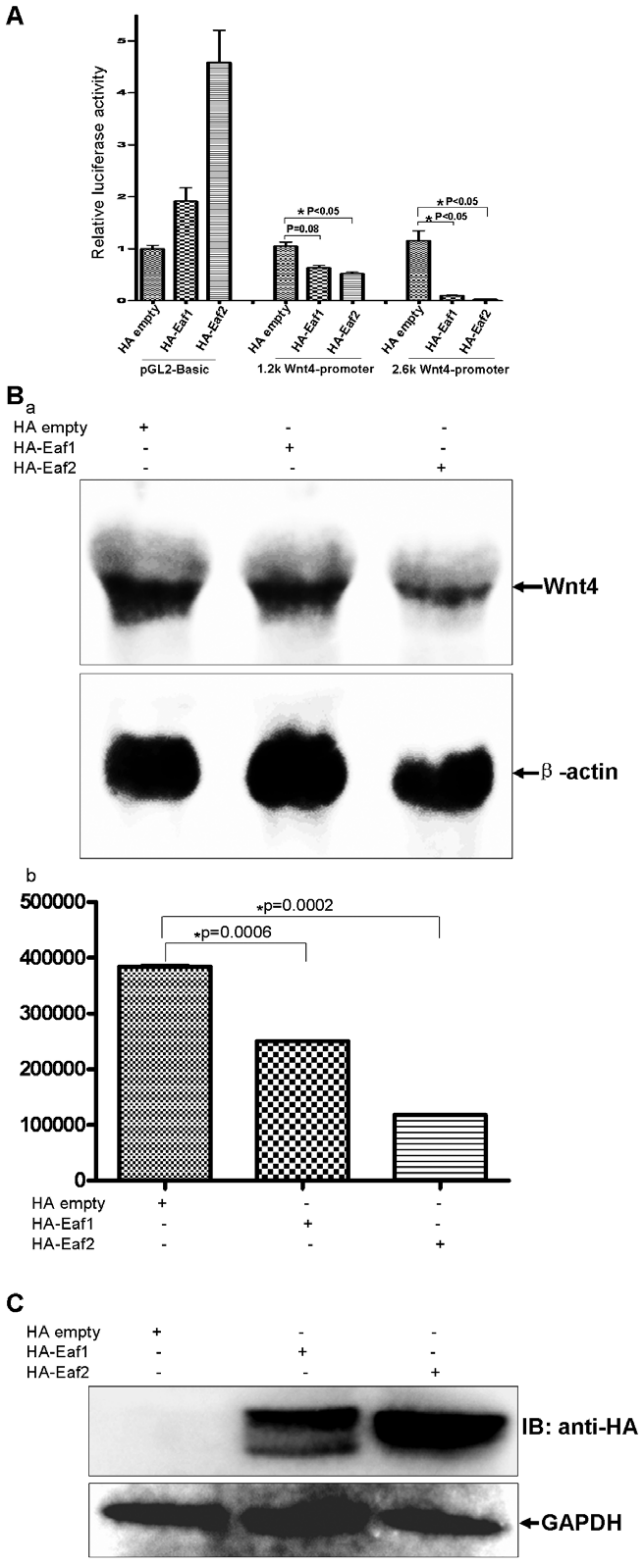
Human Wnt4 expression is suppressed by human EAF1 and EAF2/U19 in a human cell line. (**A**) 293 cells were transfected with human *Wnt4* promoter (1.2 kb and 2.6 kb)-reporter constructs and a control vector or vectors expressing EAF1 or EAF2/U19. Luciferase activity was normalized to *Renilla* and reported as the mean ± SD. (**B**) (a) Endogenous Wnt4 expression in 293 cells is suppressed by the over-expression of either EAF1 or EAF2/U19 as revealed by Northern blot analysis. (b) Northern blots were quantitated and then normalized to β-actin mRNA.

Of note, EAF1 and EAF2/U19 are nuclear proteins that harbor transactivation domains [1], [3], [4], [9]; thus, we hypothesized that EAF1/2 might be able to act as transcriptional repressors for wnt4a, despite the fact that these proteins possess transactivation domains. To test this possibility, we performed *Wnt4*-promoter reporter assays in 293 cells. 293 cells were co-transfected with the pGL2-Basic vector (control), the pGL2-1.2 kb (−1228− −36) *Wnt4*-promoter reporter or the pGl2-2.6 kb *Wnt4*-promoter reporter [13]coupled with the HA-empty vector (control), or vectors expressing HA-EAF1 or HA-EAF2/U19. Figure 7A shows that co-transfection of either EAF1 or EAF2/U19 inhibited activity of both the 1.2 kb and the 2.6 kb *Wnt4* promoter. The inhibition, except in the case of co-transfection of EAF1 with the 1.2 kb *Wnt4* promoter, was statistically significant (Fig. 7A). In contrast, EAF1 and EAF2/U19 induced transcriptional activity of the pGL2-Basic empty vector, ruling out the possibility of a non-specific inhibition of EAF proteins on the *Wnt4* promoter.

We next carried out Northern blot analysis to confirm the suppressive effect of EAF1 and EAF2/U19 on *Wnt4* transcription in 293 cells. As shown in Figure 7Ba and 7Bb, compared to the control, ectopic overexpression of either HA-EAF1 or HA-EAF2/U19 decreased *Wnt4* mRNA. Notably, EAF2/U19 had a greater effect, which is consistent with of the results from the promoter assays (Fig. 7A). The protein expression of HA-EAF1 and HA-EAF2/U19 was verified by Western blot analysis (Fig. 7C). Together, the data demonstrate that EAF1 and EAF2/U19 suppress Wnt4 expression in mammalian cells, perhaps in a direct manner.

### Human EAF1 and EAF2/U19 Bind to Wnt4 Promoter *In Vivo*

**Figure 8:**
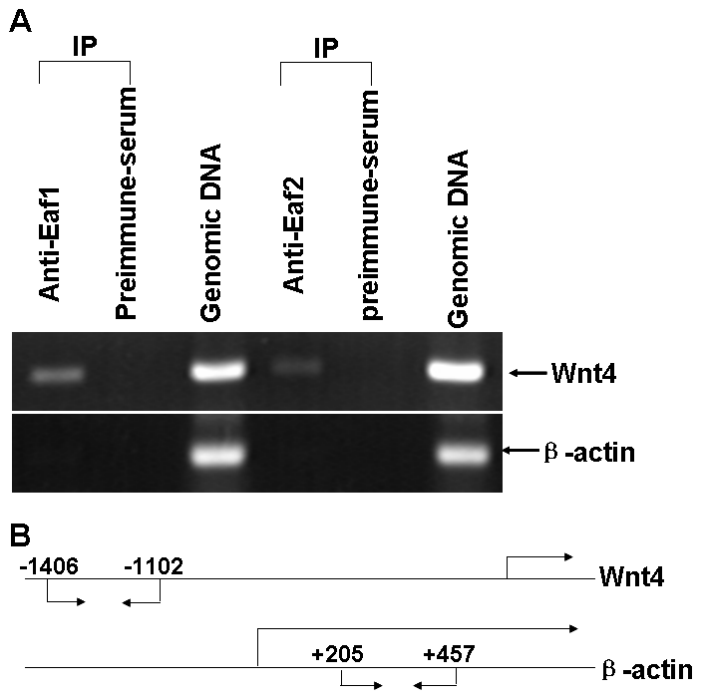
Both human EAF1 and EAF2/U19 bind to the human *Wnt4* promoter as revealed by chromatin immunoprecipitaion (ChIP) assays. (**A**) 293 cells were treated with formaldehyde to create cross-links between EAF1 or EAF2/U19 and chromatin. The chromatin was isolated, sheared, and immunoprecipitated (IP) using polyclonal antibodies against human EAF1 and EAF2/U19, or preimmune serum as control. The presence of chromatin fragments corresponding to the *Wnt4* gene or to the *β-actin* gene promoter was assessed by PCR using gene-specific primers. The gel shows the recovery of Wnt4 and actin gene fragments from the protein-chromatin input on the lane 3 and 6 (from left to right) as well as those recovered after immunoprecipitation with the anti-EAF1 antibody (lane 1), with the anti-EAF2/U19 antibody (lane4) and with the pre-immune serum (land 2 and 5). (**B**) Schematic diagram depicting the fragment of the EAF1, EAF2/U19 and actin genes that were amplified. The positions of PCR primers used to detect EAF1, EAF2/U19 and actin promoter fragments are indicated by arrows.

To test whether EAF1 or EAF2/U19 binds directly to the *Wnt4* promoter *in vivo*, we did a ChIP analysis. We immunoprecipitated cross-linked chromatin from 293 cells with an EAF1-specific antibody or an EAF2/U19-specific antibody. The precipitated chromatin was then analyzed using a primer pair that amplified a segment of the *Wnt4* promoter (−1406 to −1102) (Figure 8B). A segment of β-actin promoter amplified by a specific primer pair was used as a control. The ChIP assays demonstrated that endogenous EAF1 and EAF2/U19 proteins bound to the *Wnt4* promoter. In contrast, there was no EAF1 or EAF2/U19 protein bound to the promoter region of β-actin (Figure 8A). Thus, these data suggest that EAF1 and EAF2/U19 bind directly to the *Wnt4* promoter to modulate expression levels.

## Discussion

A previous study identified EAF2/U19 as a downstream factor in the non-canonical Wnt4 signaling important for eye development in *Xenopus laevis* [12]. In this study, through promoter assays, whole mount *in situ* hybridization, semi-quantitative PCR, Western blot analysis and embryo rescue experiments in zebrafish embryos and mammalian cell lines, we provide additional data supporting EAF2/U19, as well as EAF1, as downstream factors of Wnt4. Although we did not provide direct evidence to show that EAF1 is a downstream factor of non-canonical Wnt4 signaling, the possibility exists that this is the case based on the conserved structure and function between EAF1 and EAF2/U19.

The role of Eaf2/U19 in embryogenesis appears to be different among species. In mouse, *Eaf2/U19* gene-targeting did not cause any detectable defects in embryos [10]. This is not the case in other species: in *Xenopus laevis*, eye development requires EAF2/U19 [12] while in zebrafish, Eaf2/U19 knockdown resulted in a fusion of the eyes (cyclopia), probably due to a C & E movement defect [19]. The possibility exists that *Eaf2/u19*-knockout mice do not show detectable defects in embryogenesis due to a redundancy between Eaf1 and Eaf2/U19, but at the same time, we have evidence that in zebrafish, Eaf1 and Eaf2/u19 are not fully redundant [19]. Clearly, more studies are required. Even so, the results from our studies in zebrafish and mammalian cells as well as studies done in *Xenopus* cells suggest that the regulation of EAF1 and EAF2/U19 by Wnt4 may be conserved across species, even if the functional roles of the proteins are not.

**Figure 9:**
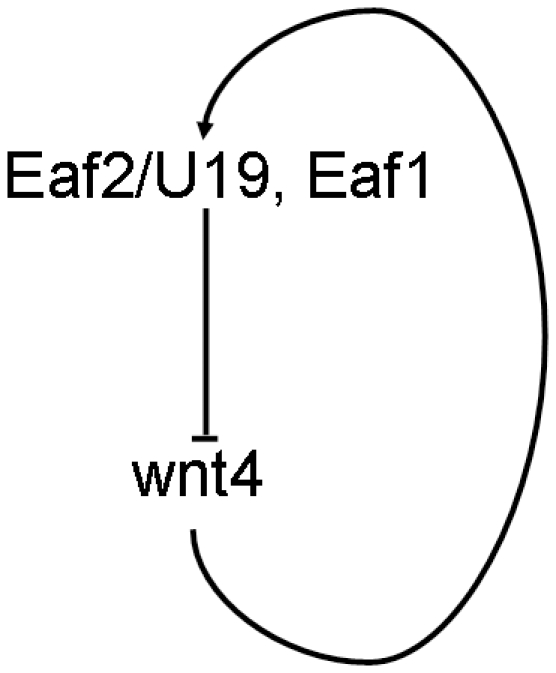
Schematic diagram of the negative feedback regulation loop between Wnt4 and the Eaf gene family.

In addition to Wnt4 regulating EAF1 and EAF2/U19 expression, we also found that EAF1 and EAF2/U19 bound directly to the *Wnt4* promoter in 293 cells, causing suppression in Wnt4 expression. These observations led us to propose that Eaf and Wnt4 form an effective auto-regulatory feedback loop to maintain an appropriate level of expression (Fig. 9). Based on the analysis of zebrafish embryos and 293 cells, this auto-regulatory negative feedback loop may be evolutionary conserved.

Notably, both EAF1 and EAF2 have been shown to serve as positive regulators for ELL in transcriptional elongation revealed by *in vitro* assays [8]. Here, we provide evidences to show that EAF gene family could also act as transcriptional repressors for directly inhibiting Wnt4 expression by *in vitro* and *in vivo* assays. Although the roles of EAF gene family in gene regulation are poorly understood so far, regarding we have showed previously that EAF gene family could up-regulate non-canonical wnt signaling ligand *wnt11* in zebrafish embryos [19], it implied that EAF gene family might play important roles in regulating gene expression either serving as activator or serving as inhibitor. However, the mechanism underling these activities needs to be further characterized.

Although we identified a regulatory relationship between Wnt4 (Wnt4a) and both EAF family members in zebrafish as well as mammalian cells, we also noted differences. While Wnt4 (Wnt4a) up-regulated EAF expression in both zebrafish embryos and 293 cells, the effect was more pronounced in the 293 cells. This suggests that other factors may modify the ability of Wnt4 to induce expression. The difference in factors may originate from using an *in vivo* model versus a tissue culture model or may be species specific. In addition, we observed a difference between EAF1 and EAF2/U19: Wnt4 more strongly induced EAF2/U19 expression, which in turn more strongly suppressed Wnt4 expression. This implies that the auto-regulatory loop formed between Wnt4 and EAF2/U19 is more stringent than the loop formed between Wnt4 and EAF1. In addition, even though wnt4 (wnt4a) affected the expression *eaf1* and *eaf2/u19* between zebrafish embryos and human cells in a similar way, no obvious conserved core elements in the promoter regions of either *eaf1* or *eaf2/u19* could be identified between human and zebrafish. This fact might suggest that non-canonical wnt4 (wnt4a) signals influence *eaf1* and *eaf2/u19* expression through different molecular mechanisms between zebrafish and mammalians.

EAF1 and EAF2/U19, the only two members in the EAF family [4], share a high degree of sequence homology and a conserved structure. Little is known regarding the function of EAF1, but evidence clearly supports a tumor suppressive role for EAF2/U19. Given the similarity of these proteins and their similar function in the EAF-Wnt4 feedback loop, the possibility exists that EAF1 might also play an important role in tumor suppression. Our observation that both EAF1 and EAF2/U19 participate with Wnt4a in a negative feedback loop in zebrafish embryogenesis may shed light on the tumor suppressive function of EAF proteins. Indeed, several tumor suppressive factors, including the classic tumor suppressor p53 gene family members p63 and p73, the Wilms' tumor suppressor WT1, and the cyclin/CDK inhibitor p21 regulate *Wnt4* expression [22], [23], [24]. P63, p73 and WT1 positively regulate transcription of *Wnt4*, while p21, directly downstream of Notch1 activation, negatively regulates transcription. Although to date no *Wnt4* mutations have been detected in any tumors [23], the above observations suggest that Wnt4 signaling may contribute to tumor initiation or progression and that the appropriate level of Wnt4 expression protects against cancer, highlighting the potential importance of the EAF-Wnt4 feedback loop.

Interestingly, in addition to the difference of regulatory stringency presented between EAF1-WNT4-EAF1 loop and EAF2/U19-WNT4-EAF2/U19 loop, we also noticed that the response elements in Wnt4 promoter accounting for EAF gene suppression might not be the same based on the promoter and ChIP assays. Eaf2/U19 suppressed the activity of the 1.2 kb (−1228 to -36) *Wnt4* promoter in a statistically significant manner (Fig. 7A, p<0.05) while EAF1 did not (Fig. 7A, p<0.08). In addition, EAF1 could bind to the −1406 bp to −1102 bp region of the *Wnt4* promoter much more strongly than EAF2/U19 could, as revealed by ChIP assays (Fig. 8A). Together, this data suggest that the EAF1 response element for suppression on the *Wnt4* promoter is probably localized to the region between −1406 bp to −1128 bp, while the EAF2/U19 response element is probably between −1228 bp to −36 bp. A better understanding of the functional difference between EAF1 and EAF2/U19 will help us to better define the precise roles of EAF1 and EAF2/U19 in embryogenesis and tumor suppression. However, due to the relative low activity of Wnt4 promoter reporter in 293 cells, we encountered a barrier to further define the core response elements accounting for Eaf1 or Eaf2/U19's suppression in the *wnt4* promoter. In future, to search for suitable cell lines in which wnt4 promoter reporter exhibits higher activity might be helpful for doing fine domain mapping in wnt4 promoter.

In summary, we provide evidence to showing that both EAF2/U19 and EAF1 are downstream factors of Wnt4. Furthermore, we identified a novel auto-regulatory negative feedback loop between Wnt4 and the EAF gene family. Although the molecular mechanisms underlying this loop require further definition, this study has shed new light on the targets of non-canonical wnt4 (wat4a) signaling as well as has opened a new window for understanding the mechanism of the EAF gene family in tumor suppression.
